# Diapause and quiescence: dormancy mechanisms that contribute to the geographical expansion of mosquitoes and their evolutionary success

**DOI:** 10.1186/s13071-017-2235-0

**Published:** 2017-06-26

**Authors:** Diego Felipe Araujo Diniz, Cleide Maria Ribeiro de Albuquerque, Luciana Oliveira Oliva, Maria Alice Varjal de Melo-Santos, Constância Flávia Junqueira Ayres

**Affiliations:** 1 Entomology Department, Aggeu Magalhães Institute, Oswaldo Cruz Foundation, Av. Professor Moraes Rego, s/n – Cidade Universitária, Recife, PE Brazil; 2 Zoology Department, Federal University of Pernambuco, Av. Professor Moraes Rego, 1235 – Cidade Universitária, Recife, PE Brazil

**Keywords:** Culicidae, Seasonality, Metabolism, Adaptation, Dispersion, Disease transmission

## Abstract

Mosquitoes are insects belonging to the order Diptera and family Culicidae. They are distributed worldwide and include approximately 3500 species, of which about 300 have medical and veterinary importance. The evolutionary success of mosquitoes, in both tropical and temperate regions, is due to the various survival strategies these insects have developed throughout their life histories. Of the many adaptive mechanisms, diapause and quiescence, two different types of dormancy, likely contribute to the establishment, maintenance and spread of natural mosquito populations. This review seeks to objectively and coherently describe the terms diapause and quiescence, which can be confused in the literature because the phenotypic effects of these mechanisms are often similar.

## Background

Mosquitoes are arthropods that can cause considerable nuisance and affect human health worldwide [1, 2]. They are among the most prolific and invasive species, contributing to the spread of endemic diseases [3, 4]. These organisms are present in most places on the planet, from the Arctic to the most remote desert oases, except Antarctica due to its extremely low temperatures. Thus, mosquitoes are widely diverse and can easily be found in a wide variety of habitats, including forested, rural and urban environments [2, 5].

These insects have been intensely studied since the end of the nineteenth century due to their ability to act as hosts for many pathogens, including helminths, protozoans and arboviruses, that cause disease in humans and other vertebrates [2, 6]. However, only 10% of the approximately 3500 mosquito species are medically relevant [1, 7–11].

Mosquitoes, especially from the genera *Anopheles*, *Aedes* and *Culex*, include vectors for three major groups of human pathogens: parasites from the genus *Plasmodium*, which cause malaria; filarial worms from the genera *Wuchereria* and *Brugia*; and many arboviruses, including the agents of dengue, yellow fever, chikungunya, zika and others [12–14]. Estimates by the World Health Organization (WHO) indicate that diseases transmitted by mosquitoes are among the major causes of morbidity and mortality in developing countries [15], and high densities of mosquitoes severely challenge vector control programs [16]. The explosive growth of natural mosquito populations is strongly related to the survival and dispersion strategies that some species have acquired over the course of their evolutionary history [17].

Dormancy is a biological trait that may play an important role in the maintenance of natural populations and refers to a physiological phenomenon characterised by the interruption or reduction of metabolic activity in an organism. In mosquitoes, dormancy can occur at different stages of the life-cycle [18]. Diapause and quiescence represent different types of dormancy found in many species of mosquitoes. In this review, these terms are analysed for their conceptual principles and their respective delayed developmental effects; in addition, the mosquito species found to exhibit these phenomena will be noted.

### Insect dormancy and its various types

Dormancy is a physiological phenomenon defined as a state of suspended development or suppressed metabolic activity in an organism [19]. Dormancy can occur in both plants and animals; in insects, it can manifest in the embryonic (pharate larvae), immature (larvae and pupae) and adult stages [18]. This phenomenon can be triggered by climactic signals, especially the photoperiod for temperate climate insects and relative humidity for tropical insects. This adaptation seeks to promote survival during and after unfavourable environmental conditions and is known in the literature as heterodynamic development [20]. In 1869, the term dormancy was first described as a period of inactivity caused by low temperatures by the French researcher Duclaux*,* who was studying silkworms (*Bombyx mori*) [20, 21]. According to a literature review by Danks [20] on the definitions and terminology of dormancy in insects, dormancy is divided into two major categories: diapause and quiescence. The terms diapause and quiescence have been reported to be synonymous in the literature [8, 22–27], but these survival strategies arise from distinct signalling pathways even though the strategies have the same goal: to ensure survival during and after environmental stress.

Mosquitoes belong to one of the most well-adapted taxa in the insect group; they are present across most of the planet, they occupy diverse niches and are potential disease vectors [2]. Diapause and quiescence are well characterised in several stages of the mosquito life-cycle. In the embryonic phase, for example, both strategies have the same effect: the inhibition of larval hatching. Conversely, only diapause drives dormancy in the larval and adult stages of mosquitoes [28].

### Diapause in mosquitoes

Diapause is a well-studied seasonal survival strategy and is influenced by several factors, such as the species-specific ecological interactions, biogeography, life history and physiology of many insects [29]. The etymology of the word “diapause” comes from the Greek *diapausis* (pause), derived from the verb *diapauein*, which means to stop or to decrease activity at a time of constant action [30]. Biologically, Tauber et al. [31] defined the diapause phenomenon as a dynamic state of low metabolic activity that is genetically determined and mediated by neurohormones that phenotypically affect individuals by decreasing morphogenesis, blocking reproduction and metamorphosis, and increasing tolerance to extreme environmental conditions

The first studies on diapause in mosquitoes coincided with early studies of seasonality, diapause and photoperiod in other insects [17]. Early reviews on the topic were performed by Lees [32], Danilevskii [33], Tauber et al. [31] and Danks [20]. Studies at the time were motivated by the mosquito’s hematophagous habit, which is linked to its ability to transmit the causative agents of several diseases such as malaria, filariasis, and many arbovirus infections (yellow fever, Western equine, St. Louis and Japanese encephalitis, and West Nile fever) [34].

Diapause is common in insects and other arthropods, especially in areas with harsh winters. Many aspects of diapause are critical for understanding the transmission cycle of vector-borne diseases, as this survival strategy contributes to the maintenance, establishment, growth and dispersion of natural vector populations after the end of an unfavourable season to their development [29]. The process of diapause seeks to reactivate development via external signals that control the genetic factors underlying the dormant phenotype. This can occur in several phases of the life-cycle, but most often only one developmental stage enters diapause [34].

### What is the environmental signal that induces diapause in mosquitoes?

Species exhibiting the phenotypic plasticity to undergo diapause have the required information encoded in their genomes. The major stimuli inducing diapause in natural populations are changing photoperiod (short days and long nights) and gradual decreases in temperature [31, 35–40]. Mosquito species that use photoperiod to signal diapause include *Aedes albopictus*, *Aedes atropalpus*, *Aedes sollicitans*, *Aedes taeniorhynchus*, *Culex pipiens* and *Culex restuans* [38, 39, 41–44].

Preparation for diapause occurs in mosquitoes when pupae and/or adult females, which are thought to be the determining stages for this biological trait, are stimulated by exposure to the seasonal changes that typically occur during transitions between a favourable and unfavourable season [29, 39, 45–47]. For *Ae. albopictus*, for instance, induced females develop their offspring for diapause, which in turn present low metabolism in each life-cycle stage during the winter [48, 49]. However, for *Cx. pipiens,* the induced pupae females express diapause when they become adults [50]. Therefore, this ecological adaptation is indispensable for coordinating the growth, development and reproduction of mosquito species found in temperate zones [29].

### Ecophysiological phases of diapause

The phenomenon of diapause consists of three ecophysiological phases [51]. The first is the diapause preparation or pre-diapause phase, which corresponds to the sensitive stage in which the insect is exposed to one or more environmental signals (token-stimuli) that trigger and initiate the phenomenon in the offspring in the following season [19, 48]. In some species, this phase is favourable for the storage of energetic reserves that will be used for basal maintenance of the insect during dormancy and the reinitiation of development at the end of the process. In addition, morphophysiological, biochemical and behavioural changes can be observed in the individuals at this phase [19, 29, 51, 52]. This occurs because some mosquito species extend the developmental time of a specific life-cycle stage (delayed developmental effects) to increase their exposure to the stimulus, which is a favourable event for ensuring that the dormancy phenotype occurs in the offspring [29].

*Culex pipiens* females programmed for adult diapause have a longer larval phase, resulting in larger pupae and adults that have more lipids than their non-diapausing homologs [53]. The fat levels in females of this same species destined for diapause continue to increase significantly during the week following the emergence of the adults, reaching twice the level observed in non-diapausing females [54]. At the molecular level, this increase in energetic reserves is accompanied by an increased expression of genes associated with lipid reserve synthesis [55]. In *Ae. albopictus*, eggs in diapause are larger and contain more lipids than non-diapausing eggs, which is likely due to the increased expression of genes involved in lipid storage during pre-diapause [56].

**Fig. 1:**
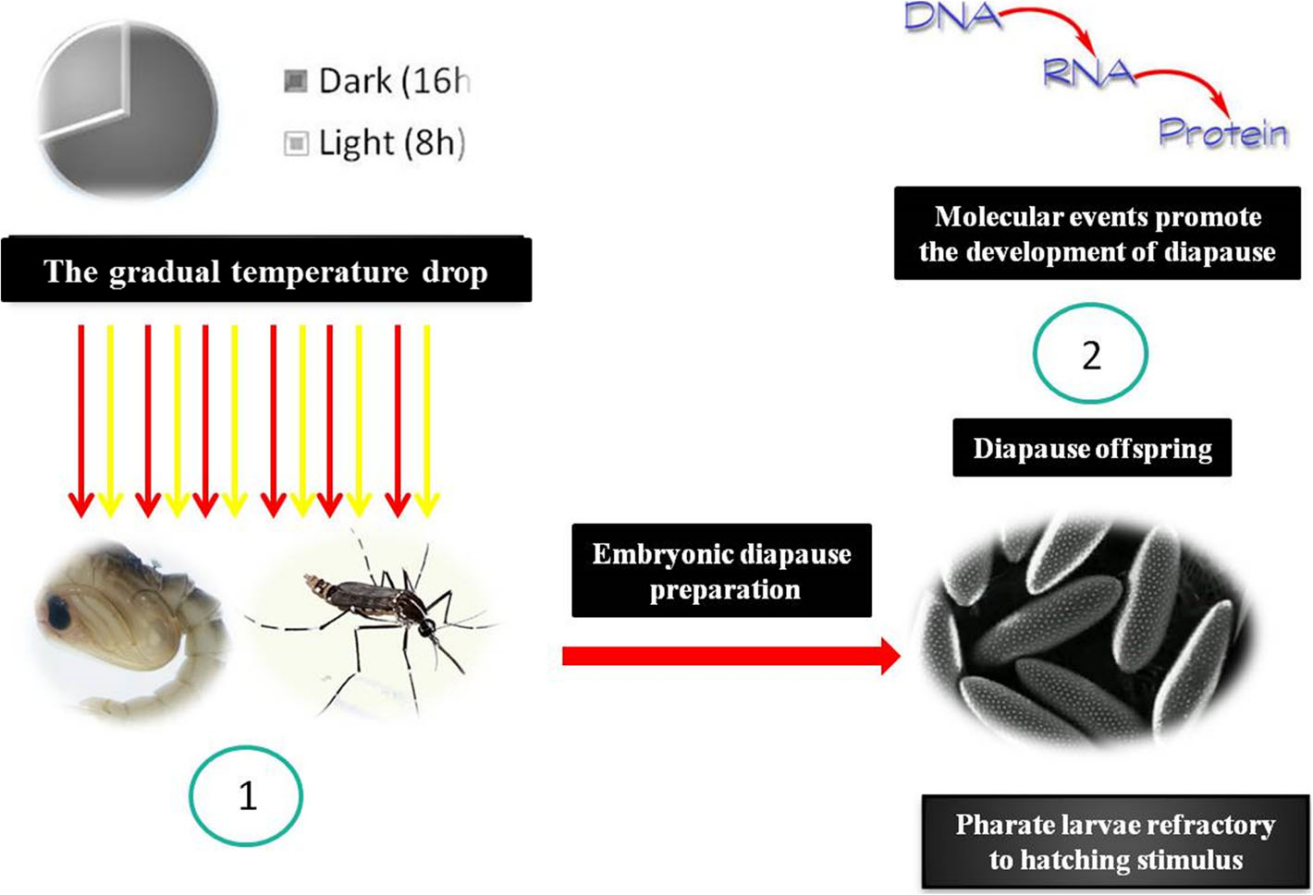
Embryonic diapause induction in mosquitoes. 1 Exposure of pupae and/or adult females to short days, long nights and gradual temperature drop, and abiotic factors that promote the preparation of the embryonic diapause. 2 Expression of specific genes transferred from the female to the offspring allows diapause to be triggered and the embryos (pharate larvae) to become refractory to the hatching stimulus

Diapause programming (Fig. 1) involves the capture of photoperiod information by the central nervous system (CNS) of gravid females, followed by a cascade of biochemical events and culminating in the transfer of a molecular diapause regulator that promotes a dormancy state in embryos [29]. Thus, clock genes can reasonably be assumed to be involved in the regulation of circadian rhythms and, consequently, in the seasonal response based on the length of day and night [57, 58]. The main clock genes in mosquitoes that are involved in circadian rhythm regulation but are not necessarily related to diapause have been characterised in *Ae. aegypti*, *Ae. albopictus*, *Anopheles gambiae*, *Cx. quinquefasciatus* and *Wyeomyia smithii* [59–65].

Diapause specifically refers to the actual time when development is interrupted or significantly slowed, and the insect does not respond to environmental stimuli [29]. This is the second phase and can be divided into the following sub-phases: (i) the responsive phase-the beginning of the process when development is stopped at a specific life stage; (ii) the initiation stage-the phenomenon is maintained and controlled by endogenous and/or exogenous factors, and (iii) termination-the time when the individuals receive the signal to return to normal metabolic activity [19]. During diapause, various endogenous changes can be observed, but these depend on the species studied. In *Ae. albopictus* embryos, *Wy. smithii* larvae and *Cx. pipiens* adults, for example, lower lipid degradation and higher tolerance to desiccation and low temperatures are present [48, 66–70].

At the molecular level, a few genetic components that mediate these adaptive physiological traits have been reported in previous studies. In *Ae. albopictus*, resistance to desiccation, promoted by diapause, results from an increase in the external surface area of the egg with increased hydrocarbon levels, and this is caused by an overexpression of a transcript involved in lipid storage. However, the mechanisms responsible for cold tolerance in this species have not been determined [71]. In *Cx. pipiens*, increased tolerance to desiccation during diapause is primarily due to an increase in the hydrocarbon layer on the cuticular body surface of adults and an increase in trehalose production, which contribute to both desiccation and cold tolerance [66]. In contrast, the molecular events that promote the effects of diapause in *Wy. smithii* have yet to be discovered [29].

**Fig. 2:**
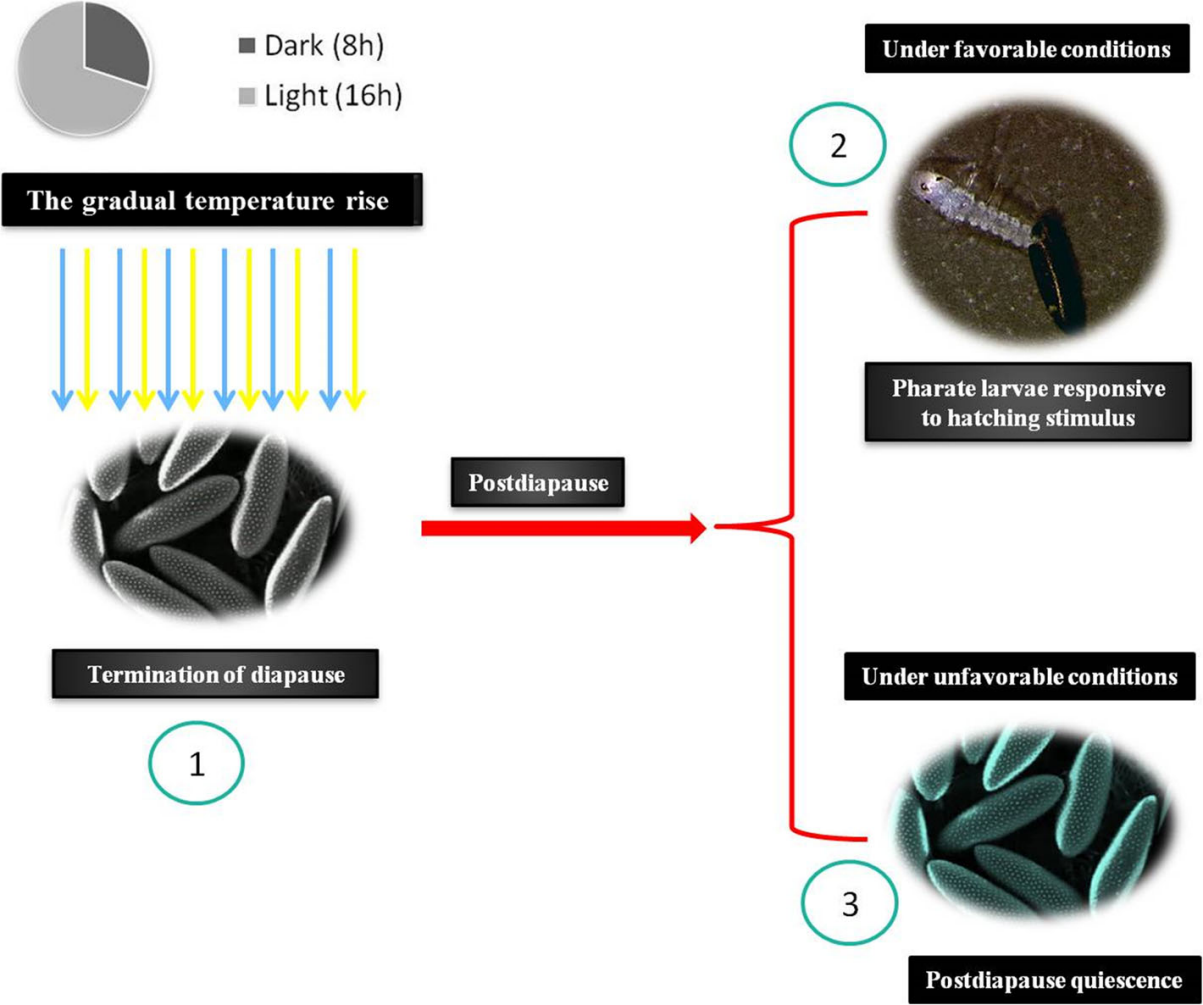
Termination of the embryonic diapause in the mosquito (post-diapause). 1 Return to normal conditions (temperature and photoperiod) that signal the end of diapause. 2 Post-diapause embryo under favorable conditions responds to the stimuli of relative humidity increase and optimal temperature in the environment, resulting in larval hatching. 3 Post-diapause embryo under non-favorable abiotic conditions is sensitive to environmental stimuli but remains dormant in a quiescent state (post-diapause quiescence) until the temperature and relative humidity become ideal for larval hatching. The dynamics of quiescence are the same as post-diapause quiescence

The last phase, termed post-diapause, is characterised by the complete reactivation of metabolism and development in the insect [51]. Although photoperiod is widely used as an environmental stimulus for entering diapause, it is less often used to signal the end of diapause; however, some exceptions exist, such as, for example, in *Wy. smithii*, where another change in photoperiod causes diapause to end [29, 72]. In *Ae. albopictus*, the termination of diapause in the eggs may be signalled by changes in photoperiod and by increasing temperature [73]. Another interesting characteristic, in addition to post-diapause, is a phenomenon known as post-diapause quiescence (Fig. 2), which is also present in *Ae. albopictus* [49, 73]. This process is considered to be a phenotypically indistinguishable phase from diapause. The insect remains in a state of dormancy, its metabolic rate continues to be low, and many of the same genes associated with diapause continue to be expressed. Thus, diapause and quiescence possibly have many molecular components in common, although the components for initial programming are exclusive to diapause [49]. Physiologically, the only difference is that during quiescence, the insect remains fully capable of responding to environmental stimuli [29, 74, 75].

### Diapause in different mosquito species/life-cycle stages

Diapause can occur in different phases of the mosquito life-cycle, i.e. in the embryo (pharate larvae inside the egg), larval and adult stages. However, this type of dormancy tends to occur in a single stage of the life-cycle for a given species [19, 29, 34]. Furthermore, in some species, diapause can occur in more than one stage, more precisely, between the embryonic and larval stages [36, 62, 76–78].

#### Embryonic diapause

This is the most common type of dormancy and occurs in the mosquito embryo. Using *Ae. albopictus,* a model organism for diapause, as a reference, the embryo is completely formed inside the egg chorion, but a metabolic depression of post-embryonic development occurs due to genetic programming; thus, the larva is unable to respond to any abiotic signals, that is, the larva is refractory to hatching stimuli [29, 45, 46, 79]. Embryos in diapause are more tolerant to desiccation and tend to have a higher total lipid content than normal embryos [57, 68, 80]. The overexpression of ecdysteroid transcripts, found by transcriptomic analysis of mature oocytes, likely regulates embryonic diapause in *Ae. albopictus* and other mosquitoes [48, 49]. The genera of mosquitoes with embryonic diapause are *Aedes*, *Anopheles*, *Psorophora* and *Ochlerotatus* [29, 34], and the major species reported in the literature for each genus are listed in Table 1.

**Table 1:** Embryonic diapause in different mosquito species

Species	References
*Aedes albopictus*	[46]
*Aedes atropalpus*	[41]
*Aedes campestris*	[150]
*Aedes canadensis*	[151]
*Aedes caspius*	[76]
*Aedes dorsalis*	[152]
*Aedes fitchii*	[153]
*Aedes geniculatus*	[77]
*Aedes hendersoni*	[78]
*Aedes hexodontus*	[154]
*Aedes impiger*	[129]
*Aedes japonicus*	[155]
*Aedes mariae*	[35]
*Aedes nigripes*	[156]
*Aedes nigromaculis*	[152]
*Aedes sierrensis*	[36]
*Aedes sticticus*	[157]
*Aedes taeniorhynchus*	[44]
*Aedes triseriatus*	[158]
*Aedes vexans*	[159]
*Anopheles walkeri*	[160]
*Psorophora ferox*	[151]
*Ochlerotatus dorsalis*	[152]
*Ochlerotatus nigromaculis*	[152]
*Ochlerotatus hexodontus*	[154]
*Ochlerotatus flavescens*	[34]
*Ochlerotatus triseriatus*	[75]
*Ochlerotatus togoi*	[75]

#### Diapause in larval stages

This physiological process is known in the literature as the syndrome of larval diapause, which is characterized in mosquitoes by the prolongation of the third- or fourth-instar. The induction of diapause in larvae is directly stimulated by a gradual decrease in environmental temperature, and the metabolic activity rapidly returns to normal in response to its normalisation in the wild, although changes in photoperiod also play a role in its induction [34]. The behaviour of the larvae is characterised by reduced locomotor and feeding activities, consequently promoting an increased accumulation of body reserves that, in turn, provide increased cold tolerance [34]. Under normal conditions, the progression of the development of the larval stages occurs biochemically through the periodic release of the steroid hormone ecdysone by the prothoracic gland, which culminates in the moults. When the larvae are in diapause, ecdysone release is lacking, and therefore, the larvae do not advance from one stage to the next [29]. Currently, no molecular studies have explained the hormonal basis for diapause in mosquitoes, but some studies have reported the absence of ecdysone as a major cause of larval diapause in other insects, which is likely similar to mosquitoes [81]. Mosquito species in which this type of dormancy has been observed are listed in Table 2.

**Table 2:** Larval diapause in different mosquito species

Species	References
*Aedes caspius*	[76]
*Aedes geniculatus*	[77]
*Aedes hendersoni*	[78]
*Aedes sierrensis*	[36]
*Aedes togoi*	[68]
*Aedes triseriatus*	[159]
*Anopheles barberi*	[161]
*Anopheles plumbeus*	[162]
*Anopheles pulcherrimus*	[85]
*Armigeres subalbatus*	[163]
*Culiseta melanura*	[164]
*Orthopodomyia alba*	[165]
*Orthopodomyia puchripalpis*	[166]
*Orthopodomyia signifera*	[167]
*Toxorhynchites rutilus*	[168]
*Wyeomyia smithii*	[169]

#### Diapause in adult females

Diapause in adult female mosquitoes involves a set of important characteristics, such as the interruption of gonadal development, reduced biting behaviour, negative phototaxis and changes in total metabolism, leading to the gradual accumulation of body fat. Mosquitoes can enter diapause in many habitats, such as caves, soil cavities, burrows, vegetable store-houses, empty sheds, basements, and catacombs [34]. In adult females, a type of dormancy occurs, known as reproductive diapause, where sexual immaturity is prolonged because the ovarian follicles do not differentiate completely and hence, delay the blood feeding activity [8, 34, 74].

The majority of studies on diapause in adult mosquitoes has been performed on the species *Cx. pipiens*, which is considered a model organism [29]*.* Under normal conditions, after the emergence of the winged form, juvenile hormone (JH) is synthesised and released, promoting ovarian follicle growth within 3 days, and shortly after, the female is ready for its blood meal, which will contribute to oocyte maturation. In contrast, females programmed for diapause do not release JH immediately after emergence, and the follicles remain atrophied. The females also have a reduced aggressiveness [82]. Experiments on diapausing *Cx. pipiens* females treated with JH have exhibited ovarian growth stimulation, confirming the importance of inhibiting this hormone to initiate diapause in adult mosquitoes [54, 83]. It is important to highlight that in this species, males do not undergo diapause, thus, they inseminate females and then die, as they cannot overwinter [31, 33, 84]. The *Anopheles* and *Culex* species reported as exhibiting adult diapause are listed in Table 3 [38, 40, 75, 85–94].

**Table 3:** Adult diapause in different mosquito species

Species	References
*Anopheles atroparvus*	[85]
*Anopheles earlei*	[86]
*Anopheles freeborni*	[87]
*Anopheles superpictus*	[85]
*Anopheles gambiae*	[88]
*Anopheles hyracanus*	[87]
*Anopheles maculipennis*	[85]
*Anopheles messeae*	[85]
*Anopheles punctipennis*	[89]
*Anopheles sacharovi*	[75]
*Culex bitaeniorhynchus*	[75]
*Culex apicalis*	[75]
*Culex modestus*	[75]
*Culex pipiens*	[39]
*Culex restuans*	[39]
*Culex tarsalis*	[90]
*Culex tritaeniorhynchus*	[91]
*Culiseta alaskaensis*	[92]
*Culiseta impatiens*	[93]
*Culiseta inornata*	[94]

### The molecular biology of diapause in mosquitoes

Most studies on the genetic basis of diapause in mosquitoes have focused on two species, *Ae. albopictus* and *Cx. pipiens*, which are considered model organisms for this approach. Early studies were performed in the fly *Drosophila melanogaster*; however, although this species is a reference for basic genetic studies, it did not yield good results in the gene expression studies, as the insect showed highly variable responses and high variance between individuals [95–97].

Diapause in *Cx. pipiens*, according to breeding experiments, is polygenetically regulated and involves genes on all three chromosomes [98, 99]. A more detailed study on the species using suppressive subtractive hybridization to determine the expression profile of diapause genes revealed that a set of 40 genes were differentially expressed. Most of these genes were implicated in the expression of structural components and responses to the environmental stress [100]. One of the upregulated genes was a stress tolerance gene expressing a heat-shock protein (HSP70), which functions as a chaperone to inhibit abnormal protein folding in harsh environmental conditions, including desiccation and cold [101]. In addition, metabolic genes are overexpressed in *Cx. pipiens* during diapause, including the mitochondrial malate dehydrogenase (*mmd*), methylmalonate-semialdehyde dehydrogenase and cytochrome oxidase (*cox*) genes. These genes may be involved in the specific metabolic events associated with diapause and have been implicated in increased cold tolerance. The expression of certain cytoskeletal genes was also upregulated by preparation for diapause. The actin gene, for example, is overexpressed during the diapause preparation stage, likely due to increased flying activity before dormancy begins, and the expression levels of this gene decrease gradually during diapause and are low at diapause termination. Downregulated genes included the ribosomal genes S3A, rpS6 and rpS24, which are involved in gene regulation (translation initiation) and inhibit or reduce the expression of several other metabolic genes [102].

Most information on changes in gene expression associated with diapause in mosquitoes is based on recent high-throughput sequencing studies (such as RNA-seq) examining the transcriptome of *Ae. albopictus* at different stages [29, 48, 49, 102]. Early studies on the differential expression of transcripts were performed in the ovary cells of this mosquito (oocytes), and later, the molecular mechanisms during embryogenesis were investigated.

A study by Poelchau et al. [48], who sequenced the oocyte transcriptome of diapausing *Ae. albopictus* females, and another more recent similar study from the same group, Poelchau et al. [49], who used diapausing embryos from *Ae. albopictus*, revealed the overexpression of genes involved in various biological processes. The following are included among these genes: the gene *ing1*, which encodes for the inhibitor of growth protein and is involved in the interruption of the cell division cycle [52, 103]; the gene *rack1*, a putative receptor for activated protein kinase C, which may bind to several signaling molecules, including transcription factors related to ecdysone (20-hydroxyecdysone), and is probably associated with the preparation for diapause [104, 105]; the gene *pepck* (phosphoenolpyruvate carboxykinase), whose product participates in the glycogen pathway to move from aerobic to anaerobic metabolism in diapausing mosquitoes [106, 107]; and the gene GPCR (G protein-coupled receptor), which is involved in increased resistance to environmental stress [108].

### Quiescence in mosquitoes

Quiescence is a type of irregular dormancy (non-seasonal) characterised by slowed metabolism and directly resulting from unfavourable environmental conditions, including low humidity and high temperatures [22, 74, 109, 110]. This adaptive trait is often confused with diapause, especially when referring to embryonic dormancy, but quiescence is a less complex biological trait that does not depend on endogenous control for its initiation. Stimuli that trigger quiescence are referred to as acyclic environmental changes [19]. Quiescence also differs from diapause because it is neither a previously programmed event, nor is it hormonally controlled; once the stimulus that induces the process ceases, physiological activity is restored [29, 34, 73]. Because quiescence is controlled exogenously, it is possible that rapid gene activation and macromolecule synthesis or degradation are not required for entry into the quiescent state [109].

In mosquitoes, as in other organisms, the term quiescence is applied to various biological events. Most commonly studied in the egg, quiescence in mosquitoes can be stimulated in different stages or structures, enabling the insect to attain favourable conditions for survival. In the mosquito *Cx. quinquefasciatus*, for example, mature spermatozoids are maintained in quiescence in the male reproductive tract and are activated in response to specific chemical signals [111]. In this species, motility is stimulated by substances from the accessory glands in males and is possibly controlled by protein phosphorylation and Ca^2+^ levels [111]. In addition, in females, degenerative dilations may develop in the ovary, which contains granular material during winter, and the presence of these expansions in the ovaries is thought to be indicative of quiescence [112, 113].

In the family Culicidae, quiescence, unlike diapause, has been primarily observed in the egg, reflected in the resistance to desiccation that allows the embryo to survive in dry conditions. The process begins when the embryo (pharate larvae) receives an external stimulus, such as a rapid drop in humidity or change in temperature, which signals unfavourable environmental conditions and impedes larval hatching [19, 34, 74]. In this case, the developmental arrest is temporary and immediately reversible, as contact with water induces rapid hatching; that is, the quiescent embryo is not refractory to hatching stimuli as is found in diapausing embryos [18, 19, 34, 49, 114]. As shown in Table 4, the genera reported exhibiting quiescence are *Aedes*, *Anopheles* and *Culex* [23, 49, 68, 80, 115–124]. The species *Ae. aegypti* is prominent among mosquitoes due to its strategy of prolonged viability by embryonic quiescence, significantly contributing to the constant expansion of populations in the wild [74, 75]. However, several studies have erroneously reported this trait as diapause [8, 22–24, 26].

**Table 4:** Embryonic quiescence in different mosquito species

Species	References
*Aedes aegypti*	[68]
	[23]
	[116]
	[170]
	[118]
	[119]
	[120]
	[121]
	[122]
	[115]
	[123]
*Aedes albopictus*	[68]
	[117]
	[71]
	[48]
*Aedes flavopictus*	[68]
*Aedes galloisi*	[68]
*Aedes riversi*	[68]
*Anopheles aquasalis*	[121]
	[123]
*Anopheles gambiae*	[124]
*Culex quinquefasciatus*	[123]
	[123]

### Egg quiescence or embryonic desiccation resistance

Egg quiescence is commonly referred to as “embryonic desiccation resistance” (EDR) and depends on several factors that range from differences in eggshell composition and structure to physiological changes, resulting in reduced metabolism in the larvae contained within the egg [22, 116, 121, 125]. However, because the ability to resist desiccation is a property of the egg and not of the embryo and because desiccation can occur at other stages of development, the term “egg resistance to desiccation (ERD)” has been suggested as more appropriate for referring to this phenomenon [123].

The three layers that form the eggshell, the exochorion, endochorion and serosal cuticle, are particularly important for ERD [116, 123]. The first two layers are produced in the ovary, by females, and are, therefore, present at laying [74, 123, 126]. The serosal cuticle (the innermost layer), in turn, is an extracellular matrix produced by the extraembryonic serosa during early embryogenesis. In *Ae. aegypti*, secretion of the serosal cuticle occurs between 11 and 13 h after oviposition and approximately 8 h post-fertilization in *An. gambiae* [115, 124].

This cuticle likely secretes a chitin-containing material under the chorion, the external layer of the egg, making it impermeable and protecting the embryo from desiccation [116, 123]. Changes in the amounts of the eggshell components are associated with water loss regulation and are fundamental for determining the intensity of egg dehydration. *Aedes albopictus* females exposed to short day length in temperate regions produce eggs in photoperiodic diapause, unlike populations in tropical regions, which enter quiescence. One of the characteristics of the egg that permits this adaptation is the high quantity of fatty acyl-CoA elongase in the tissue of mature oocytes responsible for producing hydrocarbons in the eggshell [71]. These hydrocarbons regulate water loss in insect eggs, and the abundance of this enzyme varies in the eggs of *Ae. albopictus* exposed to long and short days in temperate populations but is maintained at relatively constant levels in tropical populations [80, 123]. In addition to several hydrocarbons in the eggshell, the amount of chitin is another factor involved in ERD in mosquitoes, such as *Cx. quinquefasciatus*, *An. aquasalis* and *Ae. aegypti*. Eggshells with higher amounts of chitin are more resistant to desiccation [123].

### Quiescence patterns in container-inhabiting mosquitoes

ERD has been more commonly studied in container-inhabiting mosquitoes, including *Ae. aegypti* and *Ae. albopictus*. In urban areas, females often lay their eggs in containers with clean water, especially disposable containers, tires, plant pots and water storage containers [127, 128]. Because the eggs are laid near the water surface, this developmental phase is very susceptible to dehydration, particularly during the first few hours of development [129].

First-instar larvae that remain inside quiescent eggs have been referred to as pharate first-instar quiescence [34, 74]. Normal development finishes approximately 3 days after oviposition and larval survival depends on maternal reserves [119]. Throughout the quiescent period, the larval developmental period is significantly prolonged, and lipid reserves are reduced, incurring fitness costs for larval viability, compromising the reproductive performance of the adult [34, 74].

Minimally studied, quiescence in mosquito eggs does not appear to have a uniform pattern, exhibiting variability between species or even among populations of the same species [69, 123, 130]. Under similar low-moisture conditions, the pharate first instars of *Cx. quinquefasciatus*, *An. aquasalis* and *Ae. aegypti* can survive for a few hours, 1 day or several months, respectively [123]. These differences may be due to traits inherent to the eggs of each species, such as size, the structure of the exochorion and endochorion, differences in metabolite quantity and formation of the serosal cuticle [68, 121, 131, 132].

Brazilian colonies of *Ae. aegypti* maintained at a temperature of 28 ± 1 °C, a relative humidity of 80 ± 5% and a photoperiod of 12 h had a viability period of up to 492 days, with high hatching rates between three and 121 days [23]. A similar pattern with high larval hatching rates (80%) was reported by Diniz et al. [115] in quiescent *Ae. aegypti* eggs that had been stored for up to 150 days. The authors compared eggs from laboratory and wild populations with different susceptibilities to the insecticide temephos, which were then maintained for up to 180 days at 26 °C, with a photoperiod of 12 h and at 50–60% humidity. The high viability of quiescent eggs from temephos-resistant females suggests a high contribution to the maintenance of resistant individuals in the wild. Similarly, in Australia, quiescent *Ae. aegypti* eggs remained viable for more than a year with a hatching rate of approximately 2–15%, allowing its dispersion to new locations [133]. Species inhabiting forests have been shown to be less resistant to changes in humidity. *Aedes riversi*, *Ae. galloisi* and *Ae. flavopietus* eggs have different survivability rates in very humid conditions but were less resistant than *Ae. aegypti* and *Ae. albopictus* under low humidity. Intraspecific differences in ERD were also observed among these species, as *Ae. riversi* and *Ae. flavopietus* strains from subtropical regions had lower viability than strains from temperate regions [68].

### The molecular biology of quiescence in mosquitoes

Although much is known about the metabolic mechanisms and molecular biology of diapause, very little is known about these aspects during quiescence in mosquitoes. A study by Poelchau et al. [49] that compared the transcriptomes of quiescent and diapausing *Ae. albopictus* eggs found that the genetic expression profile between these samples converged over time; that is, the transcription profile in eggs during late diapause (40 days) is similar to that in quiescent eggs [49]. An important aspect of this study is that expression levels of genes related to lipid metabolism were always higher in eggs in diapause, demonstrating the likely importance of this reserve for maintaining embryonic diapause and explaining why eggs in diapause have more lipid reserves than quiescent eggs [49].

Currently, the metabolic pathways or hormones associated with quiescence are unknown, and only the chitin synthase (CHS) gene has been described as being related to this phenomenon in mosquitoes. This gene promotes the synthesis of chitin, which is then secreted into the extracellular space of the egg, with direct implications for the formation of the serosal cuticle and consequently the resistance to desiccation [116, 124, 134]. Despite being primarily cited for *An. gambiae* (AgCHS), this gene is highly conserved in two other species of mosquitoes, *An. quadrimaculatus* and *Ae. aegypti*. The gene has two variants, but only the allele AgCHS1 is involved in embryogenesis. In *Ae. aegypti*, for example, the expression of the gene peaks between nine and 12 h after oviposition, coinciding with the acquisition of resistance to desiccation through the complete covering of the embryo by the chitinized serosal cuticle [116, 124].

### Eco-epidemiological importance of quiescence

In Europe, a considerable increase in invasive mosquito propagation has been observed since the end of the 1990s, with the species *Ae. albopictus*, *Ae. aegypti*, *Ae. japonicus*, *Ae. atropalpus* and *Ae. koreicus* already established on the continent [131]. In addition to increased population densities, the distribution of *Ae. albopictus* has continued to increase, and several other species of *Aedes* are being reported in new countries each year [135]. For example, recently, a research group from Brock University reported the detection of *Ae. aegypti* for the first time in Canada [136]. In addition, Lima et al. [137] reported a permanent *Ae. aegypti* local population in the Capitol Hill neighbourhood in Washington DC that can overwinter. This is contrary to the previous hypothesis that different introductions of *Ae. aegypti* every year maintain that local population. All these species are well adapted to the urban environment, exploiting a variety of container habitats that proliferate near human settlements, and both quiescence and diapause may be contributing to the maintenance of these populations. In addition to the annoyance of their bites, these mosquitoes are potential vectors for agents that cause tropical diseases, including Zika, dengue, chikungunya and yellow fever [138]. Quiescence in *Ae. aegypti* may also allow the survival of infected embryos, favouring virus survival and its maintenance in nature [8, 122, 139]. For example, DENV-1 was detected and isolated in 8.33% of *Ae. aegypti* eggs in Florida, suggesting that maintenance of dengue outbreaks in 2009 and 2010 in Key West may have been facilitated by vertical transmission [140]. Transovarian transmission of DENV in the field was also detected in larvae and adults originating from larvae collected in domestic containers in Rajasthan, India. Approximately 1.09% of the reservoirs contained larvae with the virus, detected by the indirect fluorescence antibody test and reverse transcriptase polymerase chain reaction. In this case, dormant eggs may have contributed to prolonging dengue epidemics [141]. Furthermore, Zika virus, a flavivirus that has recently caused large outbreaks in several countries and has been linked to microcephaly cases and other neurological complications, has also been reported as being transferred via transovarian transmission by *Ae. aegypti* and *Ae. albopictus* [142–144, 145].

Although the implications of quiescence on the ecology of mosquito vectors and public health are well established, its effects on physiology, behaviour and life history are less understood. Maternal reserves accumulated in eggs directly influence the period of dormancy in the first-instar larvae contained within the eggs [8, 120]. Thus, quiescent eggs pose an important problem for vector control because these eggs can directly contribute to the maintenance of mosquito populations in treated areas. *Aedes aegypti* eggs from a single laying, at the same age, and maintained under the same environmental conditions had different hatching rates during the same period of quiescence. *Aedes aegypti* embryos employ a hedge betting mechanism not all eggs hatch at the first stimulus; some need a second wetting stimulus to hatch [146]. This will ensure that in the event of sudden unfavourable conditions, such as cold temperatures or a dry spell, following the oviposition of the egg batch, the entire batch is not lost [34]. Another explanation could be that not all larvae hatch simultaneously because of competition for space and resources as noted by Livdahl et al. [147] for *Ae. triseriatus*. Furthermore, Ebrahimi et al. [148] showed that the eggs of *An. gambiae* embryos are not stimulated to hatch when the water surface is agitated, demonstrating that environmental factors could indicate the best time for hatching. Sota & Mogi [68] suggested that intraspecific variation in the survival time of eggs is an inherited trait dependent on environmental pressures. Variations in the length of quiescence of eggs and variable hatching rates may be mechanisms that *Ae. aegypti* employs to produce continuous, although fluctuating, populations of adults in the wild at various stages, depending on the existence of favourable or unfavourable environmental conditions [23].

Therefore, quiescence provides a high adaptive potential to *Ae. aegypti* and *Ae. albopictus* populations, increasing the viability of their eggs and the chances of surviving in nature [148, 149]. This trait has contributed to the geographical expansion of these two species at a global level, an issue that is closely related to the spread of diseases [122].

## Conclusions

As presented in this review, dormancy, especially diapause and quiescence, has a significant impact on the life history of mosquitoes, as well as of many other arthropods. Dormancy is part of the life history of many mosquito species, providing a mechanism to overcome unfavorable seasons in tropical and temperate zones. This trait may have independently evolved several times in the family Culicidae, as the phenomenon occurs at various developmental stages in different species. These adaptive strategies provide, on an evolutionary scale, mechanisms for species survival, as offspring continue to be produced, even when exposed to the various types of stress found in a habitat, and this, in turn, contributes to the territorial expansion of natural populations, consequently increasing their invasive potential. Diapause and quiescence are not the same biological phenomenon but have been treated as synonymous in previous studies. In addition, these different types of dormancy likely aid the propagation of the transmission cycles of diseases caused by different types of arboviruses, as these etiological agents can be transferred via the transovarian route. Both of these biological phenomena could play important roles in the ecology and evolution of many insect species, such as, for example, the mosquito *Ae. albopictus*, which has both phenotypes. Thus, the phenotypic plasticity generated by these intrinsic characteristics results in the reproductive success and survival of mosquitoes in the face of adverse environmental conditions and the different control measures practised by humans. These mechanisms are also fundamental for adapting to more frequent changes in climate. These phenomena are possibly still developing and need to be more thoroughly studied, as the information generated from associated research may be applied to innovative control strategies.

## Abbreviations

AgCHS: Chitin synthase of *Anopheles gambiae*
CHS: Chitin synthase
*cox*: Cytochrome oxidase
EDR: Embryonic desiccation resistance
GPCR: G protein-coupled receptor
*ing1*: Inhibitor of growth protein
JH: Juvenile hormone
MMD: Mitochondrial malate dehydrogenase
*pepck*: Phosphoenolpyruvate carboxykinase
*rack1*: Receptor for activated protein kinase C
